# Effects of different anesthetics on remifentanil-induced postinfusion hyperalgesia in patients undergoing percutaneous endoscopic interlaminar discectomy: a randomized controlled trial

**DOI:** 10.3389/fmed.2025.1679322

**Published:** 2025-11-25

**Authors:** Yanzhe Ba, Yanan Wang, Leilei Dai, Shaozhong Yang

**Affiliations:** 1 Department of Anesthesiology, Qilu Hospital of Shandong University, Jinan, Shandong, China; 2 Department of Anesthesiology, Qilu Hospital, Cheeloo College of Medicine, Shandong University, Jinan, Shandong, China

**Keywords:** remimazolam, sevoflurane, propofol, remifentanil-induced postinfusion hyperalgesia, mechanical pain threshold

## Abstract

**Background:**

Continuous infusion of remifentanil during anesthesia potentially leads to remifentanil-induced postinfusion hyperalgesia (RPH), which may be regulated by anesthesia maintenance drugs. In this study, we investigated the effects of different anesthetics (remimazolam, propofol or sevoflurane) on RPH.

**Methods:**

One hundred and eleven patients who underwent percutaneous endoscopic interlaminar discectomy (PEID) under remifentanil-based anesthesia were randomized to one of three groups as follows: anesthesia maintenance with remimazolam (Group R), propofol (Group P) or sevoflurane (Group S). The mechanical pain thresholds of the forearm and incision area were measured using Von Frey filaments preoperatively and 24 h after surgery. Pain intensity, sufentanil consumption, side effects, and comfort and satisfaction were recorded for 24 h after surgery.

**Results:**

At 24 h after surgery, the mechanical pain thresholds around the skin incision were significantly greater in Group R [77.6 (19.7) vs. 63.7 (11.0) g, *P* < 0.001] and Group P [73.9 (15.4) vs. 63.7 (11.0) g, *P* = 0.019] than in Group S. Compared with Group S, Group R [3.9 (0.9) vs. 3.4 (0.7), *P* = 0.005] and Group P [4.2 (0.5) vs. 3.4 (0.7), *P* = 0.001] had significantly greater postoperative comfort and satisfaction at 24 h after surgery. The mechanical pain thresholds for the dominant inner forearm, postoperative pain intensity, sufentanil consumption, and side effects were similar among the three groups.

**Conclusion:**

Continuous infusion of Propofol or remimazolam attenuated RPH but not acute pain or analgesic consumption after PEID, potentially lowering the risk of chronic pain.

## Introduction

Remifentanil, an ultrashort-acting μ-opioid receptor agonist, is widely utilized in clinical anesthesia because of its predictable pharmacokinetics, and it can be given in high doses to enable effective analgesia without delaying postoperative recovery (1). However, continuous infusion of remifentanil may induce remifentanil-induced postinfusion hyperalgesia (RPH), a phenomenon associated with heightened postoperative pain sensitivity and increased risks of acute and persistent neuropathic pain (2, 3). The underlying mechanisms of RPH remain debated, and existing evidence suggests that it is related to N-methyl-D-aspartate (NMDA) receptor-mediated central sensitization and gamma-aminobutyric acid (GABA)-ergic disinhibition (4–6).

There is currently no unified standard for evaluating the RPH, and previous studies have mostly used mechanical pain thresholds measured by von Frey filaments to assess changes in pain sensation (7, 8). Furthermore, the interaction between different anesthetics and remifentanil on RPH remains insufficiently understood. Previous studies suggested that RPH was more pronounced under sevoflurane anesthesia than under propofol anesthesia, but these findings were based on subjective pain scores rather than objective mechanical pain thresholds (8). Previous research revealed that thyroid surgery patients under remifentanil anesthesia had lower postoperative pain intensity and analgesic requirements in the remimazolam group than in the propofol group in the postanesthesia care unit (PACU) (9). Recent evidence further demonstrated that continuous infusion of remimazolam alleviated opioid-induced hyperalgesia in patients undergoing laparoscopic urological surgery compared with those under desflurane inhalation anesthesia (10). Nevertheless, several studies comparing the effects of remimazolam with those of propofol on the postoperative quality of recovery have reported no significant differences in pain (11, 12).

Our study aimed to determine the effects of different anesthetics on RPH and postoperative pain in patients undergoing percutaneous endoscopic interlaminar discectomy (PEID). We hypothesized that propofol or remimazolam-based total intravenous anesthesia (TIVA) would mitigate RPH more effectively than sevoflurane inhalation anesthesia in patients undergoing PEID.

## Materials and methods

This single-center, single-blinded, prospective, randomized clinical trial was performed at Qilu Hospital of Shandong University, China. The study was approved by the Medical Ethics Committee of the Qilu Hospital of Shandong University (No. KYLL-202307-020-03) and was registered in the Chinese Clinical Trial Registry (ChiCTR2300078181, principal investigator: Shaozhong Yang, date of registration: November 30, 2023) before patient enrollment. The clinical trial was registered under the broad category of minimally invasive lumbar surgery (MILS). The specific surgical procedure conducted in this study was PEID, which is a standardized and common type of MILS. The study protocol adhered strictly to the registered design. All patients provided written informed consent before participation. The study adhered to the Consolidated Standards of Reporting Trials (CONSORT) guidelines.

### Study population

The inclusion criteria for the study were patients aged 18–65 years, with a body mass index (BMI) of 18–30 kg/m^2^; diagnosed with lumbar disc herniation; categorized as American Society of Anesthesiologists (ASA) physical status I or II; and scheduled for PEID under general anesthesia. The exclusion criteria were as follows: recent use of opioid drugs; prior lumbar surgery; diabetes, renal or hepatic insufficiency; uncontrolled hypertension, arrhythmia, psychiatric disorders, neurological disease; language impairment; inability to use a numerical rating scale (NRS) for pain; or refusal to use patient-controlled analgesia (PCA).

### Preoperative assessment

During the preoperative visit, all the patients received instructions on the use of a PCA device and were assessed for pain on an 11-point NRS. This assessment captured the baseline pain intensity caused by their lumbar disc herniation. The baseline mechanical pain threshold was evaluated with Von Frey filaments at four predetermined peri-incisional sites (within a 2–5 cm radius of the planned incision) and at three distal locations (3, 6, and 9 cm from the antecubital crease on the dominant forearm) following established methods (2). Every position was measured three times at 15-s intervals, and the mean value was calculated for statistical analysis. The mechanical pain threshold was defined as the minimum force (in grams) required to bend a Von Frey filament that was perceived as painful by the patient.

### Randomization and blinding

The patients were randomly allocated to three groups at a 1:1:1 ratio on the basis of a computer-generated random sequence with a sealed envelope. Before anesthesia, an anesthesiologist opened the patient’s envelope and prepared the study drugs needed for general anesthesia. In our study, patients and postoperative outcome assessors were blinded to the group assignment.

### Intraoperative management

Upon entering the operating room, patients were continuously monitored for heart rate (HR), electrocardiogram (ECG) parameters, peripheral oxygen saturation (SpO2), upper-limb mean blood pressure (MBP) and the bispectral index (BIS). Preoxygenation was administered (6 L/min, 100%) for 3 min, followed by remifentanil infusion (2 μg/kg over 2 min then maintained at 0.2 μg/kg/min). Anesthesia in Group P received intravenous induction with propofol (1.5–2.5 mg/kg) and rocuronium (0.6 mg/kg), followed by maintenance infusion of propofol (4 mg/kg/h). Group S underwent tidal volume inhalation induction with sevoflurane (6–8%) combined with intravenous rocuronium (0.6 mg/kg), followed by sevoflurane maintenance (2%). Group R was induced with intravenous remimazolam (0.2–0.3 mg/kg) and rocuronium (0.6 mg/kg), with maintenance via remimazolam infusion (1 mg/kg/h). Endotracheal intubation was performed when the BIS was < 60. Under volume-controlled ventilation, the oxygen intake flow was set at 2 L/min, and the respiratory rate (10–15 times/min) and tidal volume (8–10 mL/kg) were adjusted to maintain the end-tidal carbon dioxide at 35–45 mmHg. The maintenance doses for the study drugs were adjusted to maintain the BIS between 40 and 60. Core body temperature was continuously monitored and maintained with a heating blanket.

All PEIDs were performed by the same spine surgery team. After localization and puncture, a skin incision, working channel placement, discectomy, and skin closure were performed sequentially. MBP and HR were recorded at different stages. When the MBP was < 60 mmHg, additional fluid and ephedrine (5 mg) were administered. Atropine (0.5 mg) was administered if the HR was < 45 beats/min.

All patients received sufentanil (10 μg) or ondansetron (8 mg) intravenously 30 min before the end of surgery. After skin closure, all intravenous and inhaled drugs were discontinued, and the oxygen flow rate was adjusted to 6 L/min. Neostigmine (0.04 mg/kg) and atropine (0.01 mg/kg) were given to reverse residual neuromuscular block when the tidal volume of spontaneous breathing exceeded 200 ml. The time intervals between discontinuation of drugs and eye opening to verbal commands (awakening time) and extubation (extubation time) were documented. After extubation, patients were transferred to the PACU and monitored for at least 30 min.

The Ramsay score was used to assess sedation levels at 5, 10, 15, and 30 min after arrival at the PACU. The management of first postoperative pain (NRS score ≥ 4) in the PACU involved the titration of sufentanil. Once the Ramsay score exceeded 3, the patient’s peripheral oxygen saturation dropped below 92%, or the breathing rate fell below 10 bpm, the titration was discontinued. The time and total dose of the first postoperative sufentanil administration were recorded in the PACU. Moreover, all patients were treated with an electronic PCA pump containing 100 μg of sufentanil diluted in 100 ml of normal saline solution for postoperative analgesia after discharge from the PACU. With a 15-min lockout period, the device was set to deliver a basal infusion of 2 ml/h and bolus doses of 1 ml. The cumulative sufentanil consumption by PCA from 0 to 6, 6 to 12, and 12 to 24 h after leaving the PACU was documented. The NRS scores for pain at rest and after movement (coughing or turning over) were assessed at 1, 6, 12, and 24 h after surgery. The von Frey filament test was performed again at 24 h after surgery, as described previously.

The incidences of postoperative side effects, such as bradycardia, hypotension, shivering, dizziness, drowsiness, postoperative nausea and vomiting (PONV), and respiratory depression, were monitored within the first 24 h after surgery. Postoperative comfort and satisfaction were assessed by evaluating pain levels, relevant side effects, and movement limitations for 24 h after surgery.

### Outcomes

The primary outcome was the mechanical pain threshold around the skin incision at 24 h after surgery. The secondary outcomes included the mechanical pain threshold on the dominant inner forearm at 24 h after surgery, NRS scores for pain at rest and after movement, postoperative sufentanil consumption, Ramsay scores in the PACU, intraoperative hemodynamic data, use of vasoactive drugs, awakening time and extubation time, side effects, and postoperative comfort and satisfaction.

### Statistical analysis

All the statistical analyses were performed using SPSS version 26 (IBM Corporation, Armonk, NY). The normality of the data was assessed through the application of the Shapiro–Wilk test. The homogeneity of variances was verified with the Levene test. Quantitative variables are expressed as the means (standard deviations, SDs) or medians (interquartile ranges, IQRs). Categorical variables are presented as numbers (proportions). The data on the anesthesia characteristics were compared among the groups by one-way analysis of variance (ANOVA) or the Kruskal–Wallis test for continuous variables and by the Pearson χ^2^ test or Fisher’s exact test for categorical variables, as appropriate. Two-way repeated-measures ANOVA was used to analyze the mechanical pain thresholds, intraoperative hemodynamic data, and NRS scores. Data from Ramsay scores, the total dose of the postoperative sufentanil titration, and sufentanil consumption via PCA were analyzed by the Kruskal–Wallis test. The time to the first postoperative sufentanil titration and the postoperative comfort and satisfaction scores were also analyzed by one-way analysis of variance (ANOVA). Moreover, the χ^2^ and Fisher’s exact tests were used to analyze side effects. *Post hoc* comparisons were conducted via the Bonferroni adjustment. For pairwise comparisons, the Bonferroni-adjusted *P*-value is presented. A *P* < 0.05 was considered to indicate statistical significance.

In our pilot trial, the mean (SD) mechanical pain thresholds around the skin incision at 24 h after surgery in the three groups (Group S, Group P, and Group R) were 65.8 (22.6) g, 70.0 (17.3) g, and 83.3 (19.7) g, respectively. Based on the pilot data and previous studies (2), we assume that a common standard deviation of approximately 20.0 g and an average difference of 15.0 g in mechanical pain threshold are clinically relevant as they represent significant changes in sensory function. A sample size of 31 patients per group was needed to detect a significant difference (α = 0.05), with a power of 90%. Assuming a 15% dropout rate, 37 patients per group were considered for our study.

## Results

**FIGURE 1:**
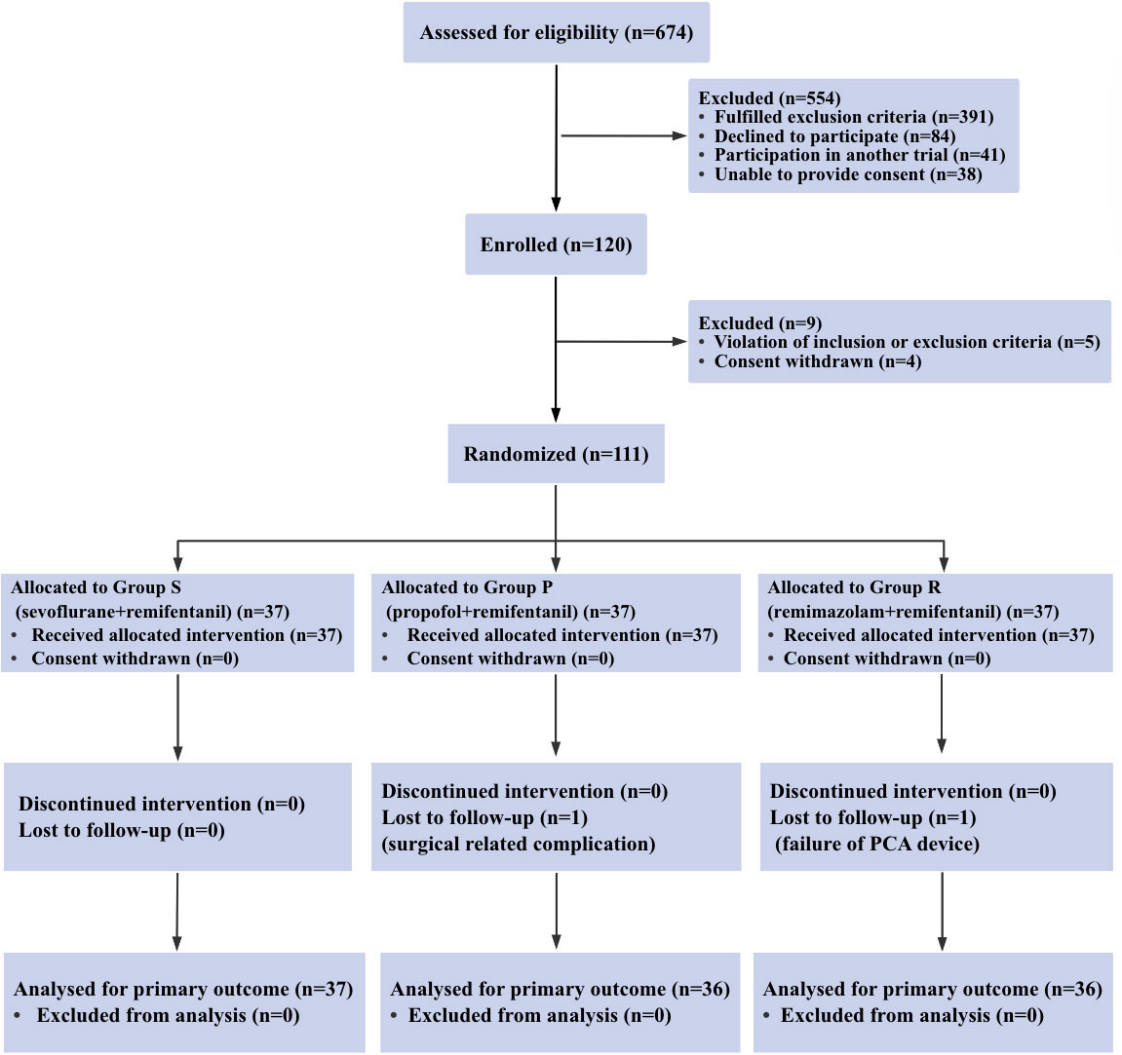
Consort diagram of patient recruitment. consort indicates the consolidated standards of reporting trials.

Between December 2023 and November 2024, a total of 120 patients were recruited for the trial, of whom 111 met the eligibility criteria, and 37 patients were randomized into each group. One patient in Group P was withdrew due to the onset of severe postoperative neurological symptoms (including lower limb and perineal numbness, and a sense of rectal tenesmus), whereas another patient in Group R withdrew because of mechanical failure of the PCA device (Figure 1). Therefore, data from 109 patients were analyzed. No clinically important differences were observed in the characteristics of the patients (Table 1).

**TABLE 1:** Characteristics of patients.

Variables	Group R (*n* = 36)	Group P (*n* = 36)	Group S (*n* = 37)
Age (y)	44.1 (11.7)	41.0 (12.5)	39.1 (10.7)
Sex (male/female)	20/16	21/15	23/14
Weight (kg)	71 (9)	73 (12)	76 (10)
Height (cm)	168 (7)	169 (8)	171 (8)
ASA status (I/II)	30/6	33/3	34/3
NRS score	5.0 (4.0–6.0)	5.0 (4.0–6.5)	5.0 (4.0–6.0)

The values are presented as the means (SDs) or medians (IQRs). SD, standard deviation; ASA, American Society of Anesthesiologists; NRS, numerical rating scale; R, remimazolam; P, propofol; S, sevoflurane.

### Intraoperative and postoperative clinical variables in anesthesia

**FIGURE 2:**
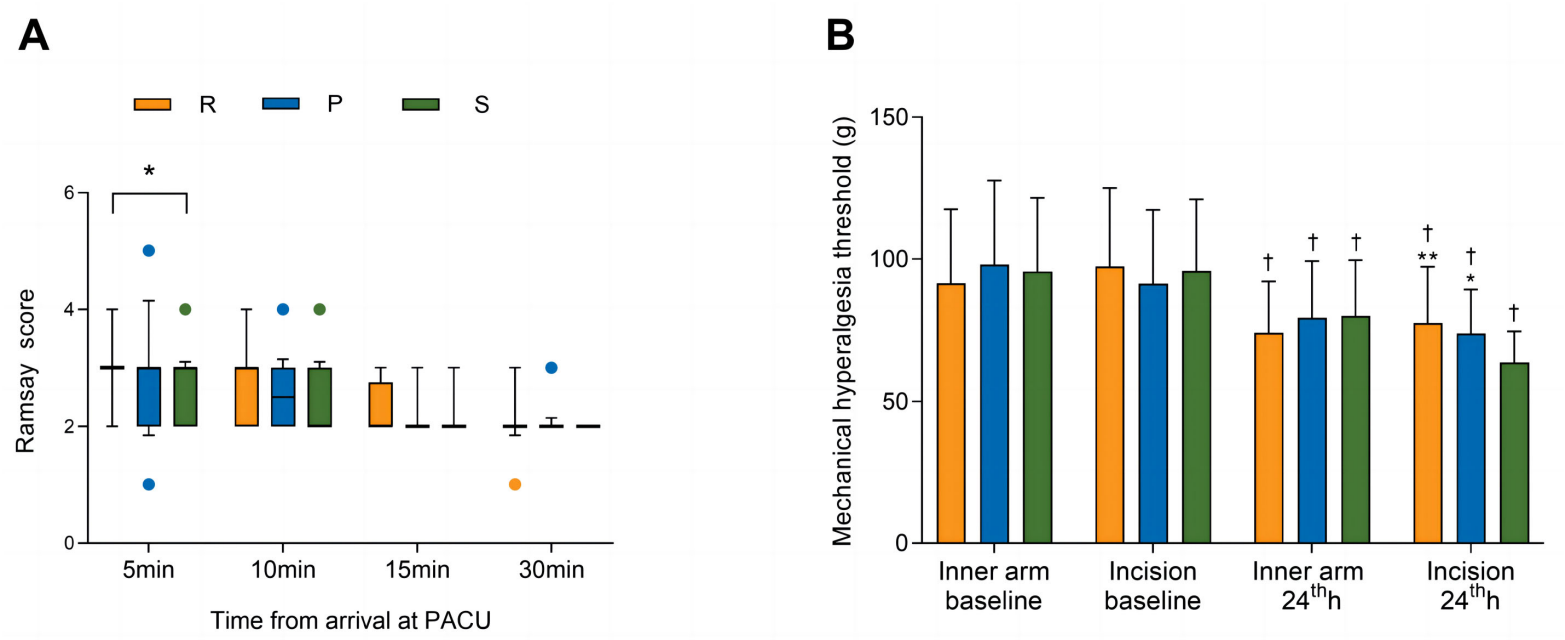
Ramsay scores in the PACU **(A)** and mechanical pain thresholds **(B).** The Ramsay scores are shown as the medians (IQRs) and were analyzed with the Kruskal–Wallis test at each time point. The mechanical pain thresholds are shown as the means (SDs) and were analyzed with two-way repeated-measures ANOVA. *Post hoc* comparisons were conducted with the Bonferroni adjustment. ^†^ *P* < 0.01 versus baseline within a group. *P < 0.05 and ^**^P < 0.01 versus Group S. ANOVA, analysis of variance; SD, standard deviation; R, remimazolam; P, propofol; S, sevoflurane.

There were no significant differences in the duration of surgery, duration of anesthesia, amount of intraoperative anesthetic drug or Ringer’s solution, or proportions of patients who were administered ephedrine or atropine among the three groups. The awakening time and extubation time were longer in Group R than in Group S (*P* = 0.022 and *P* = 0.022, respectively) (Table 2). Similarly, the Ramsay scores were higher in Group R than in Group S at 5 min after arrival at the PACU (*P* = 0.047). No significant differences were observed in the Ramsay scores among the three groups at other time points in the PACU (Figure 2A).

**TABLE 2:** Characteristics of anesthesia and postoperative sufentanil consumption.

Variables	Group R (*n* = 36)	Group P (*n* = 36)	Group S (*n* = 37)	*P*-value
Duration of surgery (min)	75 (19)	79 (24)	80 (31)	0.671*^a^*
Duration of anesthesia (min)	115 (21)	120 (25)	122 (32)	0.511*^a^*
**Amounts of intraoperative anesthetic drugs**				
Remimazolam (mg)	105 (19)	Not applicable	Not applicable	
Propofol (mg)	Not applicable	754 (194)	Not applicable	
Sevoflurane (%)	Not applicable	Not applicable	1.9 (0.4)	
Remifentanil (μg)	1500 (397)	1568 (473)	1578 (462)	0.323*^a^*
Sufentanil (μg)	10 (0)	10 (0)	10 (0)	> 0.99*^a^*
Rocuronium (mg)	45 (42–50)	45 (40–50)	45 (40–50)	0.919*^c^*
Ringer’s solution (mL)	805(178)	865(233)	895(229)	0.203*^a^*
Patients receiving ephedrine	3 (8%)	9 (25%)	6 (16%)	0.163*^b^*
Patients receiving atropine	3 (8%)	6 (17%)	8 (22%)	0.287*^b^*
Awakening time (min)	13(3)*	12 (4)	11 (3)	0.016*^a^*
Extubation time (min)	14 (3)*	13 (4)	12 (3)	0.019*^a^*
**Method of sufentanil administration**				
By titration in the PACU (μg)	0 (0–6)	0 (0–6)	6 (0–9)	0.096*^c^*
**By PCA (μg)**				
0–6 h	15.0(14.0–16.0)	15.5(15.0–16.0)	15.0(15.0–16.0)	0.195*^c^*
6–12 h	14.0(12.0–14.0)	14.0(12.0–15.0)	14.0(12.0–15.0)	0.991*^c^*
12–24 h	25.0(24.0–27.5)	26.0(24.0–28.0)	26.0(24.0–28.0)	0.293*^c^*

Values are presented as the means (SDs), numbers (proportions), or medians (IQRs). SD, standard deviation; IQR, interquartile range; PACU, postanaesthetic care unit; PCA, patient-controlled analgesia; R, remimazolam; P, propofol; S, sevoflurane. *^a^*One-way ANOVA; *^b^*Pearson χ^2^ test; *^c^*Kruskal–Wallis test; *P < 0.05 versus Group S.

### Mechanical pain thresholds

As shown in Figure 2B, the preoperative mechanical pain thresholds on the dominant inner forearm (*P* = 0.511) and around the skin incision (*P* = 0.600) were not significantly different among the three groups. Compared with those at baseline, lower mechanical pain thresholds were recorded on the dominant inner forearm (*P* < 0.001 in Group R; *P* < 0.001 in Group P; *P* = 0.002 in Group S) and around the skin incision (*P* < 0.001 in Group R; *P* < 0.001 in Group P; *P* < 0.001 in Group S) at 24 h after surgery. For the dominant inner forearm, no significant differences were found among the three groups at 24 h after surgery (*P* = 0.350). Around the skin incision, the mechanical pain thresholds for Group R and Group P were similar (*P* = 0.980) and significantly higher than that for Group S [77.6 (19.7) vs. 63.7 (11.0) g; 95% CI, 13.9 (5.0–22.9) g; *P* < 0.001; 73.9 (15.4) vs. 63.7 (11.0) g; 95% CI, 10.3 (1.3–19.2) g; *P* = 0.019, respectively] at 24 h after surgery.

### Intraoperative hemodynamic data

The MBP at each time point did not significantly differ among the three groups. No significant differences were detected in the HRs at T1 (before induction) and T7 (after extubation) among the three groups, whereas at T2 (puncture), T3 (skin incision), T4 (working channel placement), T5 (discectomy), and T6 (skin closure), the HRs in Group R were significantly greater than those in Group P (*P* = 0.010; *P* = 0.004; *P* = 0.009; *P* = 0.018; *P* = 0.014, respectively) and Group S (*P* < 0.001; *P* < 0.001; *P* < 0.001; *P* = 0.003; *P* = 0.025, respectively) (Table 3).

**TABLE 3:** Mean blood pressure (MBP) and heart rate (HR) during anesthesia.

	Group R (*n* = 36)		Group P (*n* = 36)		Group S (*n* = 37)	
Time	MBP	HR	MBP	HR	MBP	HR
T1	97 (11)	91 (18)	98 (11)	86 (13)	100 (14)	86 (13)
T2	88 (8)	78 (12)^**#^	84 (10)	68 (10)	83 (10)	68 (10)
T3	85 (8)	77 (10)^**##^	85 (10)	68 (8)	82 (10)	68 (8)
T4	86 (8)	75 (9)^**##^	82 (8)	67 (6)	81 (9)	67 (6)
T5	82 (8)	72 (10)^**#^	80 (9)	65 (8)	78 (9)	65 (8)
T6	81 (10)	68 (9)^*#^	78 (11)	63 (9)	76 (10)	63 (9)
T7	102 (10)	86 (17)	95 (18)	83 (16)	102 (14)	83 (16)

The hemodynamic data are presented as the means (SDs) and were analyzed with two-way repeated-measures ANOVA, followed by Bonferroni *post hoc* comparison. MBP, mean blood pressure; HR, heart rate; SD, standard deviation; R, remimazolam; P, propofol; S, sevoflurane. T1, baseline; T2, puncture; T3, skin incision; T4, working channel placement; T5, discectomy; T6, skin closure; T7, extubation. *P < 0.05 and **P < 0.01 vs. Group S. ^ #^*P* < 0.05 and ^ ##^*P* < 0.01 vs. Group P.

### Postoperative sufentanil consumption and pain intensity

**FIGURE 3:**
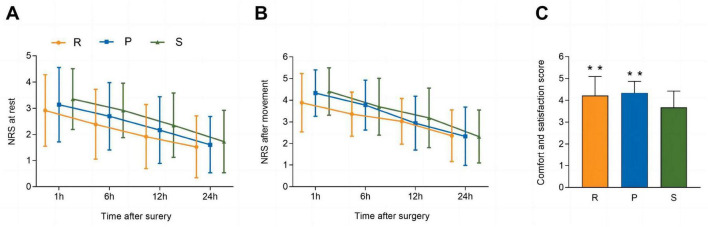
NRS scores for pain at rest **(A)** and after movement **(B)**. Postoperative comfort and satisfaction **(C)**. The NRS scores for pain at rest and after movement are shown as the means (SDs) and were analyzed with two-way repeated-measures ANOVA. Postoperative comfort and satisfaction are shown as the means (SDs) and were analyzed with one-way ANOVA. *Post hoc* comparisons were conducted with the Bonferroni adjustment. **P < 0.01 versus Group S. SD, standard deviation; PACU, postanaesthetic care unit; NRS, numerical rating scale; ANOVA, analysis of variance; R, remimazolam; P, propofol; S, sevoflurane.

In the PACU, the time to the first postoperative sufentanil requirement was longer in Group R than in Group P [14.0 (1.3) vs. 11.0 (1.7) min; 95% CI, 3.0 (0.91–5.1) min, *P* = 0.003] and Group S [14.0 (1.3) vs. 10.0 (2.6) min; 95% CI, 4.0 (2.1–6.0) min, *P* < 0.001]. No significant differences were detected among the three groups in the amounts of sufentanil titrated in the PACU or the infusion doses of sufentanil by PCA (Table 2). Regarding the pain intensity metrics, including the NRS scores at rest and after movement, no significant differences were found among the three groups (*P* = 0.210 and *P* = 0.493, respectively) (Figures 3A,B).

### Postoperative comfort and satisfaction

Table 4 shows the incidences of significant postoperative side effects. No statistically significant differences among the three groups were observed in terms of side effects, including hypotension, bradycardia, dizziness, drowsiness, shivering, PONV, and respiratory depression. However, with regard to postoperative comfort and satisfaction, patients in Groups R and P had greater satisfaction than those in Group S (*P* = 0.005 and *P* = 0.001, respectively) during the first 24 h after surgery (Figure 3C).

**TABLE 4:** Postoperative side effects.

Variable	Group R *n* = 36 (%)	Group P *n* = 36 (%)	Group S *n* = 37 (%)	*P*-value
Hypotension	3 (8)	4 (11)	2 (5)	0.632^a^
Bradycardia	1 (3)	5 (14)	3 (8)	0.238^a^
Dizziness	3 (8)	2 (6)	5 (14)	0.599^a^
Drowsiness	2 (6)	4 (11)	4 (11)	0.768^a^
Shivering	0 (0)	1 (3)	0 (0)	0.661^a^
PONV	4 (11)	4 (11)	10 (27)	0.106^b^
Respiratory depression	1 (3)	2 (6)	0 (0)	0.321^a^

The values are presented as the numbers of patients (proportions). PONV, postoperative nausea and vomiting; R, remimazolam; P, propofol; S, sevoflurane. ^a^Fisher’s exact test; ^b^Pearson χ^2^ test.

## Discussion

In this clinical trial, patients who underwent PEID and received intraoperative remifentanil infusion at 0.2 μg/kg/min exhibited RPH. Notably, the patients in the remimazolam and propofol groups presented greater mechanical pain thresholds around the skin incision at 24 h postsurgery than did those in the sevoflurane group, indicating a reduction in RPH. This discovery has significant clinical implications, as RPH may not only exacerbate acute postoperative pain but also increase the risk of chronic postoperative pain (2, 3, 13).

The current study employed remifentanil-based general anesthesia, which represents the commonly used anesthesia regimen for PEID surgery. However, previous studies have shown that patients under general anesthesia may experience increased pain after PEID due to more extensive nerve traction compared with those under local anesthesia (14). Moreover, persistent or chronic pain after spinal surgery has been reported in some patients (13), and RPH may be one of its key mechanisms. Therefore, the selection of anesthetic drugs may indirectly regulate the outcome of postoperative pain by affecting RPH.

The cellular mechanism of RPH may involve the rapid and prolonged elevation of NMDA receptor function by remifentanil, which enhances noxious stimulation signals and induces hyperalgesia (4). Previous studies have demonstrated that propofol inhibits NMDA receptors and might regulate postoperative hyperalgesia (8, 15). Similarly, sevoflurane inhibits NMDA receptors in a concentration-dependent manner (16). An animal experiment revealed that clinical concentrations of sevoflurane were insufficient to prevent hyperalgesia induced by high doses of fentanyl (17). This might explain the phenomenon of lower mechanical pain thresholds in the sevoflurane group in our study.

As a new ultrashort benzodiazepine that acts on GABA receptors, remimazolam also showed antihyperalgesia effects in our study, which is consistent with previous research results (10). The loss of inhibitory control due to the reduction in GABA levels may also contribute to the development of RPH (6), while opioid drugs can further reduce central GABA release and exacerbate sensitivity to nociceptive stimuli (7, 18). Therefore, restoring the inhibitory effect of the GABA receptor system may be a promising approach for treating RPH. Animal experiments support the ability of benzodiazepines (such as remimazolam) to reverse neuropathic hyperalgesia (19), but clinical studies have shown that their analgesic effects need to be demonstrated at higher levels of sedatives (20, 21). Our study revealed that the remimazolam group had a prolonged first sufentanil demand time after surgery, which may be related to its sedative depth or potential antihyperalgesic effect.

Previous studies have shown that noxious stimuli during surgery may have synergistic effects with remifentanil on postoperative hyperalgesia (3, 22). In contrast to the peri-incision area, the pain threshold on the dominant forearm may have less relevance to the surgical insult (2). This phenomenon might explain why there were no differences in the mechanical pain thresholds on the dominant forearm in our study.

Notably, whereas mechanical pain thresholds were significantly improved in the remimazolam and propofol groups, this improvement did not correspond to reductions in subjective pain scores (NRS) or postoperative opioid consumption, which is almost consistent with previous research findings (23). This finding is consistent with the multifaceted nature of pain, in which an objective measure of sensory hypersensitivity (quantitative sensory testing) captures a different dimension from subjective pain experience and analgesic demand, the latter being influenced by affective, cognitive, and contextual factors (24). This dissociation suggests that the primary beneficial effect of propofol and remimazolam in this context may be the amelioration of underlying opioid-induced hyperalgesia, which is a potential risk factor for chronic pain (25), rather than directly reducing postoperative pain intensity in patients with mild surgical injury. The time for the first postoperative sufentanil requirement was longer in the remimazolam group than that in the propofol group and sevoflurane group, with a trend toward less sufentanil titration in the PACU, although statistical significance was not reached. This finding might be due to a greater degree of sedation in the remimazolam group upon PACU arrival in the current study. Another reason may be the potential analgesic and antihyperalgesic effects of remimazolam. The other clinically relevant pain outcomes, such as sufentanil consumption by PCA and the NRS scores for pain at rest or after movement, did not differ among the three groups. According to previous research, there was a low correlation between the pain threshold objectively measured by Von Frey filaments and the pain intensity subjectively assessed by NRS scores (2). Compared with open surgery, surgery in our study was characterized by mild postoperative pain, which may have obscured the benefits of remimazolam or propofol for postoperative pain. The antihyperalgesic effects of these agents could be clinically relevant for more painful surgeries.

The remifentanil infusion at 0.2 μg/kg/min provided effective intraoperative analgesia and led to fewer hemodynamic fluctuations. In addition, the remimazolam group had less circulatory depression, especially in terms of heart rate, and tended to use fewer vasoactive drugs. While the remimazolam group demonstrated higher heart rates during surgical stimulation compared with the propofol and sevoflurane groups, these differences were clinically modest and did not require pharmacological intervention. This may reflect the good hemodynamic characteristics of remimazolam, allowing more appropriate physiological responses to surgical stress while maintaining hemodynamic stability (26, 27). Our results also suggested that high doses of remifentanil could be safely given with little risk of delayed recovery after surgery. The recovery times were within clinically acceptable limits in all three groups. Although longer awakening and extubation times with remimazolam than with propofol and sevoflurane were reported in our study, the differences may not be clinically relevant in daily practice.

In addition, a previous study indicated that the dose of intraoperative remifentanil administered is a risk factor for PONV (28). Propofol-based total intravenous anesthesia (TIVA) has been demonstrated to prevent PONV to a significantly greater extent than inhalational anesthesia (29). Although not statistically significant, the propofol and remimazolam groups tended to have a lower incidence of PONV compared with the sevoflurane group, which may partly explain the improvement in postoperative comfort and satisfaction. The sample size on the basis of mechanical pain thresholds may have lacked the power to detect the difference. Another adverse effect associated with remifentanil administration is postoperative shivering (30). No significant differences were observed in terms of postoperative shivering among the three groups. The use of physical heating measures during surgery may prevent the occurrence of postoperative shivering to a certain extent.

## Limitations

Our study has several limitations. First, the value of the BIS during remimazolam anesthesia was relatively high, and its accuracy has not yet been determined (31). Second, some patients with lumbar disc herniation may experience central sensitization before surgery (32), which may affect the results and limit the generalizability of the study. Third, the occurrence of chronic postoperative pain was not investigated in our study, despite the potential risk of persistent postsurgical pain associated with opioid-induced hyperalgesia. Fourth, this was a single-blind trial (patients and outcome assessors were blinded). The anesthesiologists who delivered the interventions were not blinded to group assignment due to the markedly different techniques (TIVA vs. inhalational), which may introduce a risk of performance bias.

## Conclusion

In conclusion, propofol or remimazolam infusion attenuated RPH in PEID patients. Although this did not reduce immediate postoperative pain or opioid use in our cohort with mild-to-moderate pain, mitigating hyperalgesia may lower the risk of chronic pain development. These findings warrant further investigation in more painful surgeries, where controlling RPH could yield greater clinical benefits.

## Data Availability

Data is available from the Medical Ethics Committee of the Qilu Hospital of Shandong University (Email: qlyykyc@163.com) or the corresponding author SY (contact via yszyang@163.com) for researchers who meet the criteria for access to confidential data.
